# Eliminating the Need for Anesthesia in Sleep Endoscopy: A Comparative Study of Traditional Nasopharyngoscope Design Versus NasoLens

**DOI:** 10.3390/bioengineering12060572

**Published:** 2025-05-26

**Authors:** Yen-Tsung Lin, Chih-Wei Shih, Nathan Chen, Hsin-Tzu Lu, Woei-Chyn Chu, Kuang-Chao Chen

**Affiliations:** 1 Institute of Biomedical Engineering, National Yang-Ming Chiao-Tung University, Taipei 11221, Taiwan; jason@chiyitech.com (Y.-T.L.); chinwei.be10@nycu.edu.tw (C.-W.S.);; 2 Holistic Hearing Healthcare Center, China Medical University Hospital, Taichung 40447, Taiwan; nathanchen657@gmail.com

**Keywords:** sleep endoscopy, nasopharyngoscope, image comparison, anesthesia, digital endoscope, patient comfort

## Abstract

This study investigates the potential of a novel sleep endoscope, NasoLens, to eliminate the need for anesthesia in sleep endoscopy. We assess NasoLens’ safety, maneuverability, and ability to allow sleep without sedatives, aiming to improve the overall patient experience and reduce risks associated with anesthesia. Sleep endoscopy is commonly performed under anesthesia, which introduces risks, increases costs, and can limit accessibility. NasoLens’ design aims to address these challenges by improving patient comfort and enhancing maneuverability, eliminating the need for anesthesia. This could provide a safer, more cost-effective alternative for patients, particularly those at higher risk for anesthesia-related complications. NasoLens distinguishes itself with its smaller size, teardrop-shaped head, specialized camera angle for better visualization, and an integrated microphone for real-time auditory monitoring. These features enable NasoLens to offer improved maneuverability and comfort, compared to traditional nasopharyngoscopes, while enhancing diagnostic accuracy. These design innovations could revolutionize clinical practice by minimizing anesthesia-related risks, reducing procedural costs, and improving both procedural efficiency and patient satisfaction. With its ability to allow natural sleep without sedation, NasoLens has the potential to improve patient satisfaction, procedural outcomes, and expand the feasibility of sleep endoscopy into more accessible clinical settings, making it a promising alternative to traditional models.

## 1. Introduction

Sleep-related breathing problems are widespread and cause significant negative effects on health, productivity, and overall quality of life [1]. Studies have shown that obstructive sleep apnea (OSA), for instance, is estimated to affect nearly a billion adults worldwide [2]. As these disorders can lead to other complications such as stroke and heart failure [1], it is important to diagnose the problem in order to treat it. Sleep endoscopy has become a key diagnostic tool for evaluating upper airway obstruction during sleep, particularly in patients with obstructive sleep apnea (OSA) or other sleep-related breathing disorders [3,4]. The procedure allows clinicians to directly observe the airway during sleep by using a flexible endoscope, typically inserted through the nose or mouth, to identify the sites and causes of obstruction [5,6]. Over the years, sleep endoscopy has been essential for tailoring treatment options such as surgery, positive airway pressure therapy, or other interventions [7,8].

Patient comfort during sleep endoscopy is essential to ensure a successful and tolerable procedure. Since the procedure involves inserting an endoscope into the nasal passages and throat, it can cause discomfort, anxiety, and reflex responses such as gagging or coughing, which is further exacerbated by the larger dimensions and less ergonomic designs of traditional devices. As such, the discomfort caused by the endoscope typically renders sleepy endoscopy under natural conditions to be unfeasible. Natural sleep endoscopy (NSE) has been defined as the gold standard of sleep endoscopy [4]. However, as it is labor-intensive, time-consuming, and difficult to achieve due to the patients’ discomfort, NSE has not been feasible in routine practice [4,9]. This has led to an alternative method, Drug Induced Sleep Endoscopy (DISE), to be developed as the clinical standard [4,9]. DISE is a diagnostic tool to access the dynamic upper airway collapse during sleep [9,10,11]. The sedatives used induce a state which mimics natural sleep [9,11]. By making the procedure more tolerable, anesthesia improves patient compliance, enhances diagnostic outcomes, and fosters a better overall patient experience, ensuring that necessary follow-up evaluations and treatments are more readily accepted.

However, despite its benefits in facilitating the procedure the reliance on anesthesia in traditional sleep endoscopy introduces several challenges. Although DISE appears to be practical and mimics natural sleep, the are many aspects of the procedure, such as anesthetic protocol and result interpretation, that need to be standardized [10,11,12]. As such, it is difficult to produce reliable outcomes and compare published literature [9,10]. Furthermore, although DISE mimics natural sleep, the extent to which they can be compared has not been clearly stated in studies [4,13]. This uncertainty also leads to other concerns, including ones regarding the efficacy of treatments guided by DISE. Multiple studies compare treatment outcomes after DISE and when DISE is not performed, and show that DISE does not necessarily produce better results [14,15,16]. Anesthesia itself poses significant health risks, particularly for patients with comorbidities, such as heart or lung conditions, who may be more vulnerable to its side effects, including the risk of respiratory and cardiovascular failure [17,18,19]. Inducing deep sedation in a patient with OSA may increase airway obstruction, causing a drop in oxygen saturation [20,21]. Additionally, the use of traditional nasopharyngoscopes often leads to discomfort for the patient due to their larger dimensions, making maneuverability and positioning difficult. The procedure can become prolonged, requiring deep sedation or full anesthesia to ensure immobility and comfort. This, in turn, complicates the procedure and contributes to longer recovery times and increased resource use, further heightening the risks and costs associated with sleep endoscopy. These challenges highlight the urgent need for innovative approaches that can maintain diagnostic accuracy without relying on sedation.

NasoLens is designed to address the limitations of traditional sleep endoscopy. It features minimized dimensions and a teardrop-shaped head that enhances maneuverability and ease of insertion, while a specially angled camera allows for better visualization of the upper airway. Additionally, NasoLens incorporates a microphone that provides real-time auditory feedback, synchronizing audio and video signals, improving diagnostic accuracy by allowing clinicians to assess airway sounds in addition to the image during the procedure. These design features aim to make the procedure faster, more comfortable, and less invasive, reducing the need for anesthesia. By reducing discomfort and improving maneuverability, NasoLens offers a more patient-friendly alternative to traditional nasopharyngoscopes, potentially eliminating the need for sedation or general anesthesia.

The objective of this study is to evaluate the effectiveness of NasoLens in eliminating the need for anesthesia in sleep endoscopy. On top of comparing the specifications of NasoLens to a traditional nasopharyngoscope, we assess NasoLens’ safety, maneuverability, and ability to allow sleep in patients without the use of sedatives. Our goal is to determine whether NasoLens can provide a safer, more cost-effective, and patient-friendly alternative to traditional sleep endoscopy, ultimately improving patient outcomes and reducing the risks associated with anesthesia use.

## 2. Materials and Methods

NasoLens presents a promising solution to the limitations associated with traditional nasopharyngoscopes and the use of anesthesia. Its compact design, ergonomic shape, and specialized camera angle are tailored to enhance maneuverability and patient comfort. By minimizing discomfort and improving procedural experience, NasoLens has the potential to eliminate the need for sedation entirely. This advancement not only enhances safety but also reduces costs and improves accessibility to sleep endoscopy procedures.

To evaluate NasoLens’ potential to eliminate the need for anesthesia in sleep endoscopy, three key steps were undertaken: (1) a simulation of NasoLens’ reach within the nasal canal using 3D models derived from CT scans, (2) a comparison of NasoLens’ features and traits to traditional nasopharyngoscopes, and (3) an Institutional Review Board (IRB)-approved study testing NasoLens’ ability to allow sleep in participants without the use of sedatives. The following sections describe each of these steps in detail.

### 2.1. Method of NasoLens Safety and Effectiveness Simulation

**Figure 1:**
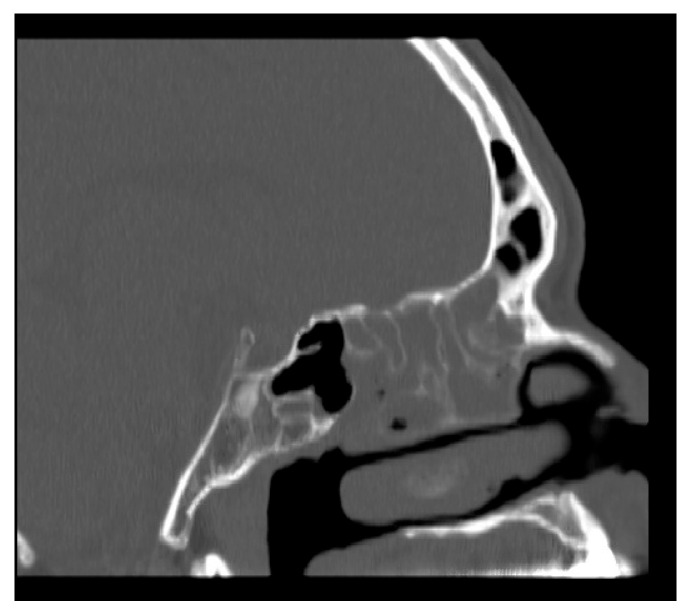
CT scan of nasopharynx.

**Figure 2:**
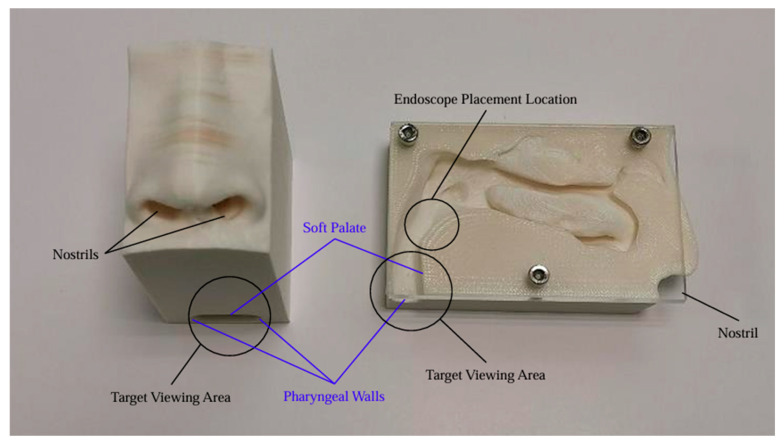
Printed 3D models of nose and nasopharynx: complete enclosed (**left**) and cross section (**right**).

We aimed to assess NasoLens’ ability to safely and effectively navigate the nasal canal without causing injury or discomfort. To simulate the nasal anatomy, a range of CT scans was obtained from a hospital. These scans were selected to capture various anatomical variations and ensure that the test models represented a diverse patient population, as shown in Figure 1. Using these scans, accurate 3D digital models of the nasal passages were generated, as shown in Figure 2. The digital models were subsequently converted into physical representations using 3D printing technology, allowing us to evaluate NasoLens’ ability to reach key target locations.

To optimize NasoLens’ design for safe and effective use, consultations were conducted with an ENT doctor. The discussions focused on determining the ideal shape, size, and camera angle that would facilitate smooth insertion and maneuverability within the nasal passages. These insights guided refinements to NasoLens’ compact design and teardrop-shaped head to enhance its compatibility with anatomical variations.

NasoLens was evaluated by inserting it into the physical 3D-printed nasal models. The procedure involved tracking the endoscope’s path to ensure it could navigate key anatomical locations in the upper airway. The tests illuminated NasoLens’ ability to avoid trauma to surrounding tissues while maintaining effective maneuverability. Comparisons were made with traditional nasopharyngoscopes to evaluate the advantages offered by NasoLens’ smaller dimensions and ergonomic design.

The success of NasoLens was determined by its ability to reach intended target locations within the nasal cavity and upper airway without any evidence of safety concerns. The trials confirmed NasoLens’ effectiveness in navigating the nasal passages, providing a potential alternative to traditional designs while addressing patient safety and comfort.

### 2.2. Method of Comparison of NasoLens to Traditional Nasopharyngoscope

To evaluate NasoLens’ performance relative to a traditional nasopharyngoscope, key design features of both devices were analyzed. NasoLens was designed with minimized dimensions and a teardrop-shaped head, intended to enhance maneuverability and reduce patient discomfort during insertion. Its specialized camera angle was optimized to improve visualization of the airway, providing a more comprehensive view compared to the traditional device.

**Figure 3:**
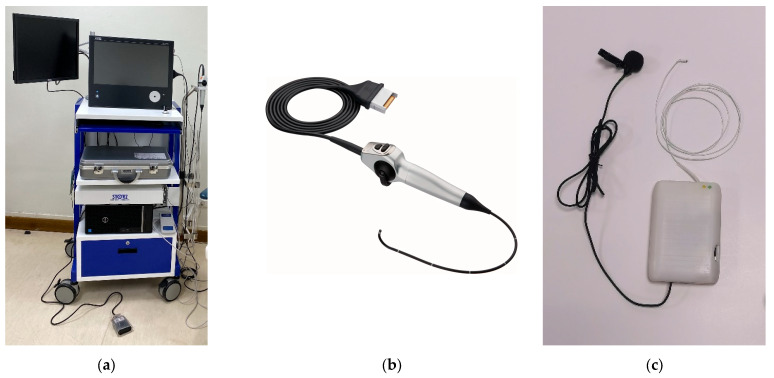
(**a**) Full system of traditional nasopharyngoscope, (**b**) the traditional nasopharyngoscope and (**c**) NasoLens, including light source, image processing, and a microphone [22].

One distinctive feature of NasoLens is its simple navigation design, which simplifies the process for the operator. In contrast, traditional nasopharyngoscopes rely on manual guidance wires, requiring more effort and precision during navigation. Traditional nasopharyngoscopes also rely on external fiber optic light sources, often recruiting Halogen or Xenon lamps, which require voluminous machines with heavy power consumption to produce sufficient illumination for endoscopy. They also utilize robust video systems, shown in Figure 3, to process the signals of the image sensor into a video signal before displaying the image onto a monitor, further adding to the bulk of external equipment. This makes the endoscope system logistically inconvenient in terms of mobility and use of space, resulting in very limited accessibility. On the other hand, NasoLens integrates not only LED lighting, but also image processing directly into the device. This design provides solid imaging with bright and consistent illumination while eliminating the need for bulky external components, allowing the endoscopic process to become portable, hence increasing accessibility.

Additionally, NasoLens incorporates an integrated microphone designed to capture and synchronize auditory signals that correlate to obstructions to the video of the upper airway—a functionality absent in the traditional device. Both devices were evaluated for their ability to navigate through the nasal passages and reach specific target locations in the airway. Special emphasis was placed on comparing maneuverability, comfort, and ease of use between the two designs.

#### 2.2.1. Image Analysis

To further assess the performance of NasoLens compared to traditional nasopharyngoscopes, a detailed image analysis was conducted for both devices. The experiments were performed in a controlled darkroom environment with backlighting to ensure consistency. A series of specialized charts was employed to evaluate specific image performance parameters. Both endoscopes were securely mounted on a holder, ensuring a stable, perpendicular orientation relative to the ground during testing.

**Figure 4:**
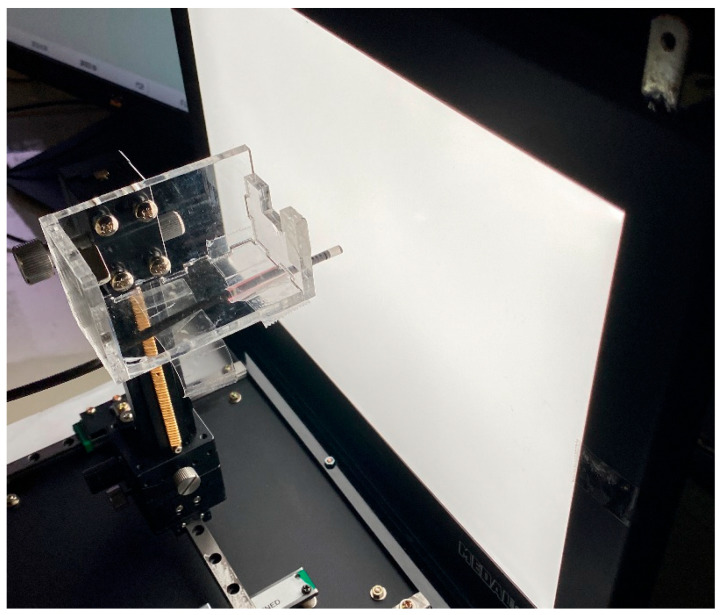
Chart photography setup. The endoscope was secured by a holder, and the lens was placed perpendicular to the image on the backlight. The distance was determined by using a fixture stand with digital calipers. After the position was confirmed, all lights, other than the backlight, were turned off, and the image was captured for analysis.

Standard charts were illuminated by an LED backlight, as shown in Figure 4, and images were captured at normal magnification without zoom adjustments. Prior to testing, white balance calibration was performed to ensure accurate color reproduction. For the visual field and distortion tests, the endoscope was placed within a multi-color temperature lightbox, positioned 40 mm from a 6500 K LED light source. A grid-patterned background was used to measure the field of view and calculate image distortion. Photographs were taken at varying distances to evaluate performance under different conditions. For all other electronic image files, MTF tests were performed using the SFRplus Chart (3nh^®^, Shenzhen City, China) positioned at distances between 3 mm and 100 mm.

Color difference was assessed using the TE188 Color Rendition Chart (3nh^®^, Shenzhen City, China), while brightness uniformity was analyzed using a gray test card placed at 20 mm. The captured images, particularly those obtained under backlighting conditions, were processed and analyzed using the Imatest software 3.7 (Imatest^®^, Boulder, CO, USA) to quantify differences in image quality. Imatest is a widely acknowledged software used for image processing, often surpassing preceding metrics in terms of accuracy and precision, and has been used in prior studies involving image analysis [23,24,25]. This setup ensured precise and repeatable evaluation of NasoLens’ imaging capabilities.

Both NasoLens and the traditional nasopharyngoscope were used to capture 30 images of standardized charts. Three investigators independently analyzed the images of the visual field and grid tests to ensure a consensus of the results. Other electronic images were analyzed using the Imatest 3.7 software (Imatest^®^, Boulder, CO, USA), a widely recognized tool for image processing in research. The analysis compares statistics of each device, including field of view, image distortion, resolution, grayscale performance, luminance, and color saturation.

#### 2.2.2. Distortion Correction

The Standard Mobile Imaging Architecture (SMIA) TV Distortion Test measures the degree of optical distortion in an imaging system by analyzing deviations from a reference grid pattern. The deviation from the standard grid chart is then presented as a positive or negative percentage, where values further from zero represent larger distortion. The software calculated the distortion ratio using the following formula:

$$r_{u}=r_{d}+k_{1}r_{d}^{3}$$

where $r_{u}$ and $r_{d}$ represent the undistorted and distorted radii, respectively [26].

#### 2.2.3. Modulation Transfer Function/Spatial Frequency Response

The Modulation Transfer Function (MTF), also known as Spatial Frequency Response (SFR), is a widely used metric in optical imaging that quantifies how well a system preserves contrast at different spatial frequencies. It provides insight into an imaging system’s ability to reproduce fine details. The modulation of the captured image can be determined by calculating the ratio of the fundamental frequency component to the DC (Direct Current) component, expressed as:

Modulation (f)=Fundamental frequency component/DC component

The resolution can be converted using the following formula:

$$N_{f}=\frac{\mathrm{picture}\;\mathrm{height}\;(\mathrm{mm})}{\mathrm{pixel}\;\mathrm{size}}$$

*N_f_* denotes the resolution limit and adheres to the Nyquist sampling theorem [26].

#### 2.2.4. Color Difference

To compare the color reproduction accuracy of NasoLens and the traditional nasopharyngoscope, a color difference analysis was conducted using a standardized 24-patch GretagMacbeth ColorChecker (3nh^®^, Shenzhen City, China). This test evaluates how accurately an imaging system captures and reproduces colors by measuring deviations from a reference standard [24]. Accurate color reproduction is essential in endoscopic imaging to ensure the proper identification of tissues and pathological features. The Imatest 3.7 software (Imatest^®^, Boulder, CO, USA) uses CIEDE2000 formulas, which are widely acknowledged to be the superior metric for measuring color differences due to their accuracy. Delta E ($\Delta E_{ab}^{*}$) was calculated using the following equation:

$$\Delta E_{ab}^{*}=\sqrt{{\left(L_{2}^{*}-L_{1}^{*}\right)}^{2}+{\left(a_{2}^{*}-a_{1}^{*}\right)}^{2}+{\left(b_{2}^{*}-b_{1}^{*}\right)}^{2}}$$

Delta C ($\Delta C^{*}$) was calculated using the following equation and follows a similar pattern:

$$\Delta C^{*}=\sqrt{{\left(a_{2}^{*}-a_{1}^{*}\right)}^{2}+{\left(b_{2}^{*}-b_{1}^{*}\right)}^{2}}$$

where $L^{*}$ denotes luminance, $a^{*}$ represents color on a green–red scale, and $b^{*}$ signifies color on a blue–yellow scale. Unlike $\Delta E_{ab}^{*}$, $\Delta C^{*}$ does not include luminance difference in its calculation [26].

#### 2.2.5. Luminance Uniformity

This assessment creates a contour plot to determine the luminance uniformity of the image [26]. Luminance is a measure of the perceived brightness of an image and can be derived from RGB (Red, Green, Blue) values using a weighted sum that accounts for human visual sensitivity to different colors. Luminance is calculated as 0.2125R + 0.7154G + 0.0721B [26]. The weights of each color correspond to the sensitivity of the human eye to that color. The values each assign a maximum value of one and correspond to a pixel level of 255 for image files with an 8-bit depth or 65,535 for those with a 16-bit depth [26].

#### 2.2.6. Grey Scale

A grayscale test chart was used to evaluate the ability of NasoLens and the traditional nasopharyngoscope to accurately reproduce different levels of brightness. The OECF chart consisted of 20 grayscale patches ranging from pure black to pure white, allowing for a detailed analysis of contrast and tonal gradation. As required by the Stepchart module of the Imatest analysis software, the camera distance to the chart was adjusted to ensure an approximate horizontal resolution of 50 pixels per patch.

#### 2.2.7. Statistics

The data underwent processing using SPSS 21 (IBM, New York, NY, USA). The data for SMIA TV distortion, SFR, color difference, luminance, and grayscale were input into the Imatest software for analysis. The average value was computed and subjected to an independent Student’s T test between two groups, with a *p*-value of <0.05 considered statistically significant.

### 2.3. Method of IRB Testing of NasoLens’ Ability to Allow Sleep

The final phase of the study involved a trial approved by the Institutional Review Board (IRB) to evaluate NasoLens’ ability to facilitate natural sleep without the use of sedatives or anesthesia. The study was under CMUH112-REC-2-153 and was conducted by the ENT department at China Medical University Hospital. The trial included 11 volunteers who provided informed consent prior to participation.

**Figure 5:**
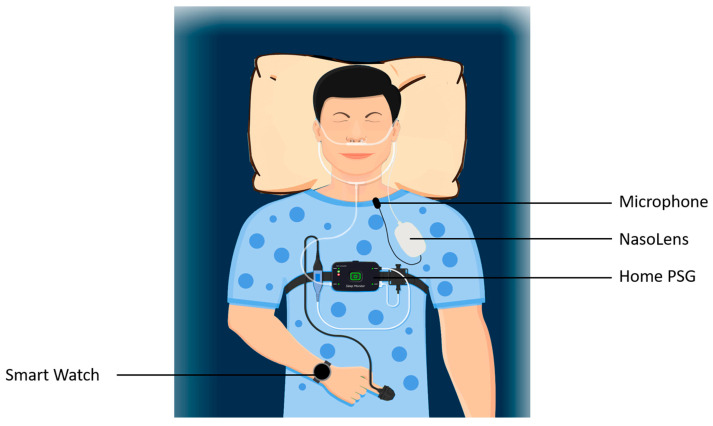
Diagram of IRB experiment setup. The subject was fitted with a Garmin watch, home PSG, and NasoLens. The subject was then instructed to attempt to fall asleep under natural conditions. The Home PSG was the ApneaLink Air manufactured by Resmed^®^ (San Diego, CA, USA). The model of the Garmin watch was the Vivoactive 5, and all participants used software version 4.76.13 of the Garmin-provided application.

**Figure 6:**
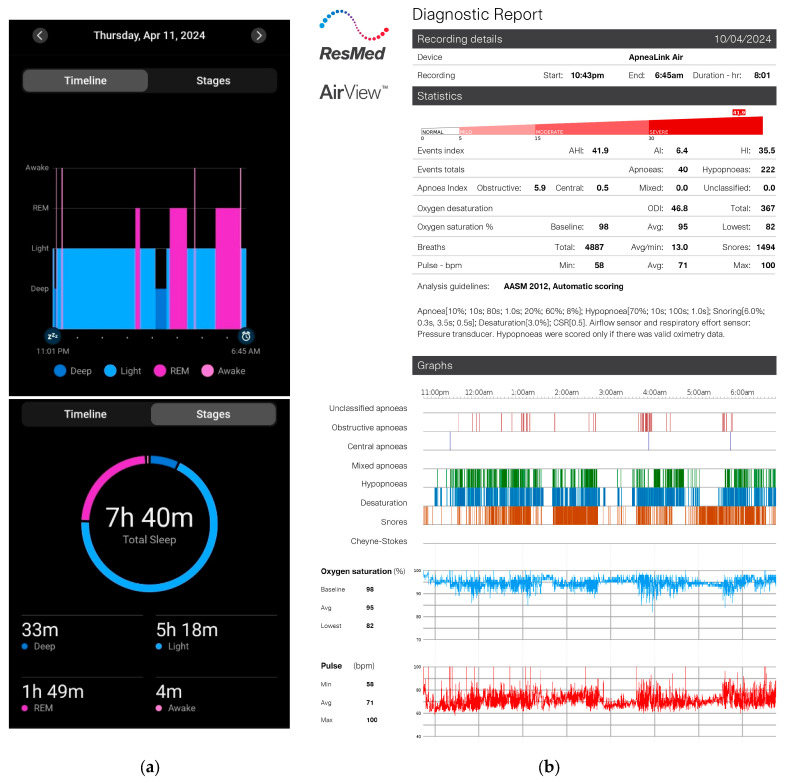
(**a**) Garmin watch report displaying a chart indicating onset of sleep and time of wakefulness, and showing amount of time spent in deep sleep, light sleep, REM sleep, and awake stages. (**b**) ResMed Home PSG report, primarily indicating AHI-related events, blood-oxygen saturation, and pulse. The above figure displays the corresponding results of the same subject on the same day. The report generated by the Garmin Watch displays the date when the individual wakes up, whereas the home PSG displays the date when the recording begins. It was observed that individuals typically had sleep onsets between 15–30 min after starting the recording on the PSG. The time of wakefulness on the Garmin Watch and the end of PSG recording are matched. Between the 11 subjects, the mean and standard deviation of AHI was 13.87 ± 10.46, the oxygen saturation percentage was 94.4 ± 0.9, and the pulse (beats per minute) was 62.55 ± 7.49.

Each participant was fitted with NasoLens and monitored for signs of sleep onset using home polysomnography (PSG) and a Garmin watch, as shown in Figure 5. The PSG setup included airflow monitors, effort sensors, position sensors, and a pulse oximeter to record sleep-related physiological parameters comprehensively. Furthermore, the PSG also reported quantitative measures with a focus on apnea-hypopnea index (AHI) related events, blood-oxygen saturation, and pulse, shown in Figure 6. We observed abnormal changes in these parameters from the home PSG results, and observed the corresponding videos during these variations. The Garmin watch featured a sleep monitor which showed whether or not an individual fell asleep. Participants were educated by an ENT physician to perform self-insertion of NasoLens. The placement was validated by the physician to ensure optimal positioning, and secured with a fixator to prevent displacement during sleep. Participants were then instructed to self-equip NasoLens at home, and attempt to fall asleep while wearing NasoLens without requiring the presence of a clinician.

The primary objective was to assess the number of participants who could naturally fall asleep while using NasoLens and to evaluate whether its design supported a comfortable sleep experience. Data collected from the PSG system were analyzed to determine sleep onset, quality, and overall compatibility of NasoLens with natural sleep processes.

## 3. Results

### 3.1. Result of NasoLens Safety and Effectiveness Simulation

**Figure 7:**
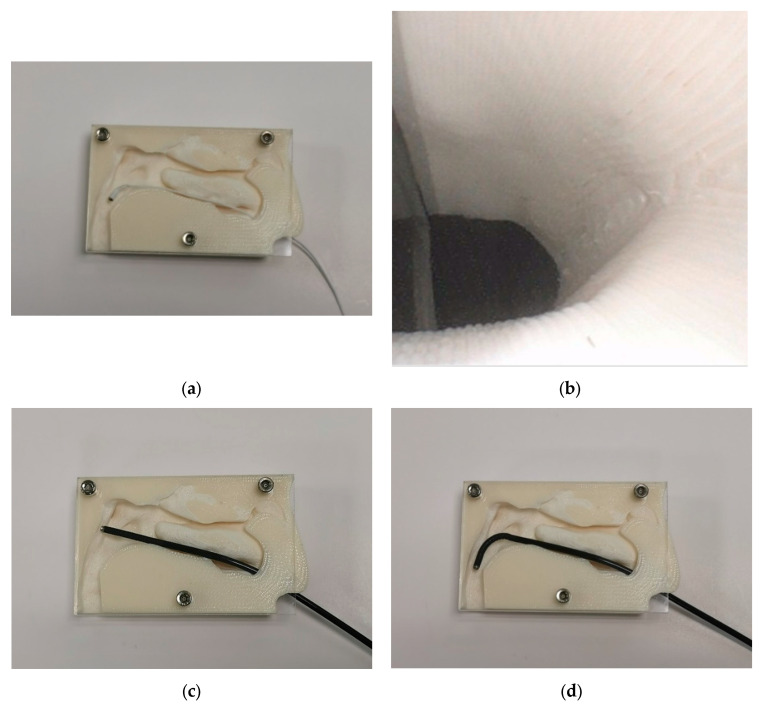
(**a**) NasoLens at target location inside nasopharynx model and (**b**) viewing angle. (**c**) Traditional nasophayngoscope inside nasopharynx model without manual turning and (**d**) with manual turning.

The simulations using 3D models derived from CT scans demonstrated that NasoLens was capable of safely reaching the target locations within the nasal canal. The endoscope’s smaller dimensions and flexible design allowed it to navigate the anatomical structures without compromising the ability to capture clear and accurate images (Figure 7). Additionally, NasoLens’ smaller dimensions and ergonomic design facilitated easier placement and reduced the extent of contact with sensitive structures, such as the nasal mucosa and the posterior pharyngeal wall.

### 3.2. Result of Comparison of NasoLens to Traditional Nasopharyngoscope

NasoLens demonstrated superior maneuverability compared to the traditional nasopharyngoscope in both the 3D model and live subjects, thanks to its smaller size and teardrop-shaped head. These design elements allowed NasoLens to navigate the nasal passages more easily, requiring less force and reducing contact with sensitive tissues. The simple navigation design in NasoLens further enhanced its ease of use, offering smoother and more precise control over the device’s movement. In contrast, the manual guidance wire of the traditional endoscope often resulted in increased effort and occasional discomfort for the patient during insertion. NasoLens’ integrated LED light source provided consistent, bright illumination without the need for external fiber optics, making the procedure more efficient and reducing equipment complexity. Both devices provided clear imaging; however, NasoLens’ ergonomic design allowed for better positioning, and its wider field of view, on top of its specialized camera angle, improved overall visualization. The inclusion of the microphone in NasoLens allowed for real-time monitoring of ambient sounds, adding another layer of diagnostic capability that was absent with the traditional nasopharyngoscope. Overall, these features, listed in Table 1, collectively facilitate a more effective and comfortable procedure.

These features collectively enhance patient comfort by reducing the scope’s invasiveness, improving maneuverability, and ensuring high-quality imaging, all of which help reduce the need for anesthesia and make the procedure more tolerable for a wider range of patients.

#### 3.2.1. Image Analysis

The image analysis demonstrated that NasoLens outperformed the traditional nasopharyngoscope in its field of view, distortion, and grayscale. The NasoLens matched performance of the traditional nasopharyngoscope in color difference, and struggled with resolution and luminance, primarily due to limitations of its minimized dimensions. These differences, nevertheless, were consulted with an ENT practitioner and determined to be acceptable during practice.

#### 3.2.2. Field of View

**Figure 8:**
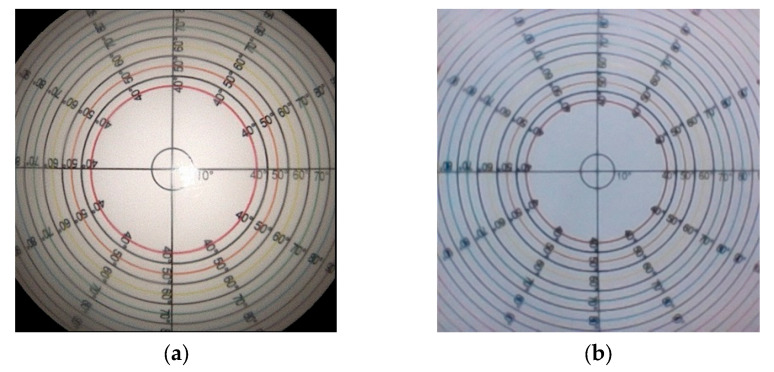
Field of view chart image captured using (**a**) traditional nasopharyngoscope and (**b**) NasoLens (right). Each concentric circle on the chart represents a different field of view angle.

The field of view (FOV) is a critical optical parameter that defines the observable area captured by the endoscope’s imaging system. A larger FOV allows for broader spatial coverage, enabling clinicians to visualize larger anatomical structures and surrounding tissues, which is particularly advantageous as orientation and context are crucial in endoscopy-related procedures. Digital endoscopes produce a rectangular or square visual field, in contrast to optic endoscopes, which display circular visual fields. Digital endoscopes are featured with three primary measurements: horizontal, vertical, and diagonal field of view [26]. The NasoLens featured a maximum field of view of 120 degrees, while the traditional nasopharyngoscope displayed 100 degrees, as shown in Figure 8.

#### 3.2.3. Distortion

**Figure 9:**
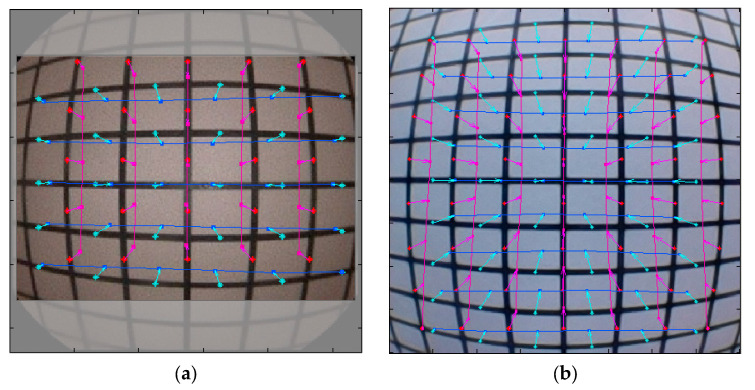
Distortion of (**a**) traditional nasopharyngoscope and (**b**) NasoLens. The pink line represents the vertical deformation, while the light green line represents the horizontal deformation. The traditional nasopharyngoscope displayed greater image distortion than NasoLens.

The endoscopes were used to perform a grid ground test and analyzed for distortion. The ratio of distortion was −11.3 ± 0.72% for NasoLens and −13.8 ± 0.66% for the traditional nasopharyngoscope (Figure 9). This indicates lesser image distortion for NasoLens (*p* < 0.001), despite a larger visual field.

#### 3.2.4. Modulation Transfer Function (MTF)/Spatial Frequency Response (SFR) Test

**Figure 10:**
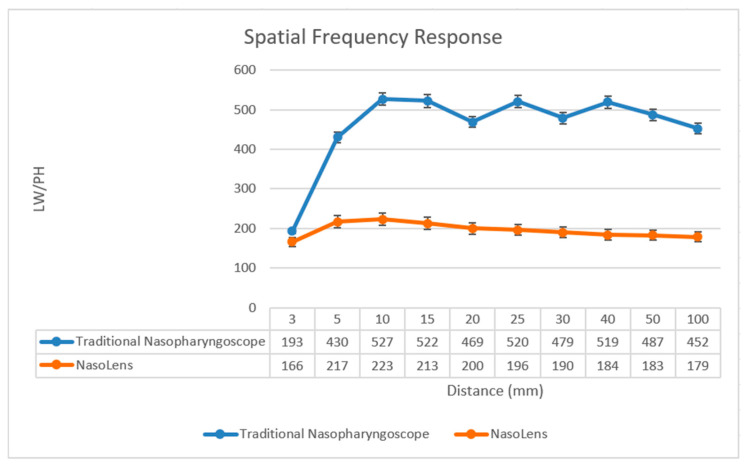
This graph displays the spatial frequency response (SFR) of both endoscopes, with the *x*-axis denoting distance (mm) and the *y*-axis representing LW/PH.

MTF and the SFR charts are essential tools for evaluating an imaging system’s ability to capture fine details and maintain contrast at different spatial frequencies. SFR charts quantify how sharply an image transitions from dark to light regions, helping to assess edge clarity and detect optical aberrations. The SFR chart images were captured across distances ranging from 3 mm to 100 mm (Figure 10). The resolution of the endoscope at different distances can be measured by the ratio of line width to lines per height (LW/PH) [26]. The findings indicated that the traditional nasopharyngoscope possessed superior resolution to NasoLens. This outcome conformed with expectations, as NasoLens’ minimized dimensions required a much smaller camera module, resulting in lower resolution. However, after consulting with an ENT professional, the image resolution in both endoscopes was found to be acceptable during practice, despite the variance.

#### 3.2.5. Color Difference

**Figure 11:**
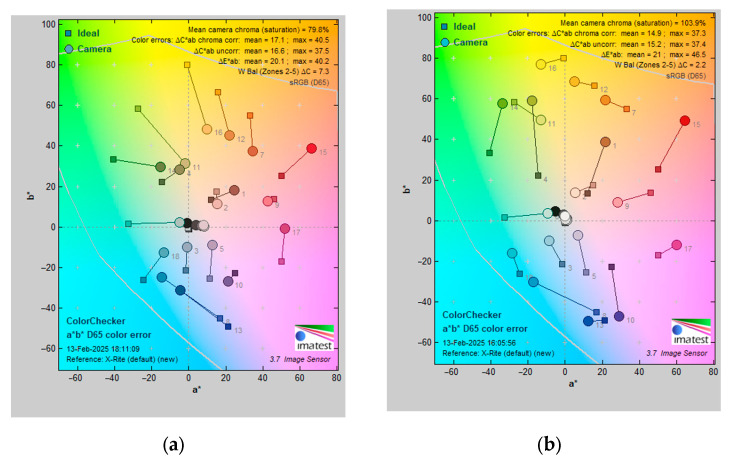
The color difference between the (**a**) traditional nasopharyngoscope and (**b**) NasoLens. In the figure, the *x*-axis represents a*, indicating color on a scale ranging from green to red, while the *y*-axis represents b*, signifying color on a scale from blue to yellow [26]. Each gray number on the chart represents a different color group, with squares representing the original colors of the chart, while circles represent the colors displayed by the endoscope [26]. The NasoLens displayed a similar level of color difference as the traditional nasopharyngoscope. The overlapping circles in the middle of the diagram represent the color differences in grayscale, which cannot be clearly depicted in the image. A separate discussion on ‘grayscale analysis’ is presented in another paragraph in Section 3.2.7.

The Color Difference Test evaluates an imaging system’s ability to accurately reproduce colors by measuring deviations from a reference standard. ΔE provides a value for overall color difference, considering variations in both chromaticity and luminance, while ΔC measures the difference in chromaticity between two colors, excluding the consideration of luminance. The traditional nasopharyngoscope exhibited a ΔC of 16.6 ± 0.44, whereas NasoLens recorded 15.2 ± 0.35. In terms of ΔE, the traditional nasopharyngoscope registered 20.1 ± 0.53, while NasoLens showed 21.0 ± 0.51 (Figure 11). NasoLens has been shown to outperform traditional endoscopes in the absence of luminance, exemplifying that NasoLens compensates for the color difference through other optical techniques. Overall, the color difference of NasoLens was on equal footing to that of the traditional nasopharyngoscope (*p* < 0.001).

#### 3.2.6. Luminance

**Figure 12:**
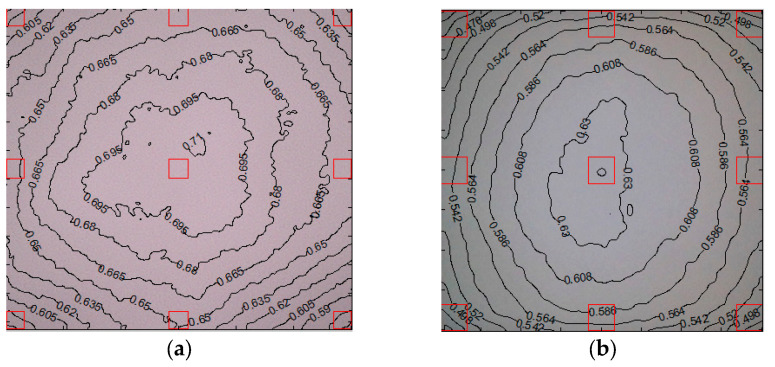
Luminance contour plot for the (**a**) traditional nasopharyngoscope and (**b**) NasoLens. The red rectangles represent the four corners, four sides, and center of the image. The black lines indicate contour areas where luminance is identical, as shown in the values corresponding to each line.

Backlight images were captured for both endoscopes, and a luminance contour plot was generated (Figure 12). The luminance contour plot was generated by displaying normalized pixel-level contours for the luminance channel of the image file. The plots display the average corner values in unnormalized pixel levels as a percentage of the maximum [26]. The mean value was 75.6 ± 2.74% for NasoLens, and 82.3 ± 2.32% for the traditional nasopharyngoscope. This indicates that the LED lights in NasoLens exhibit a less uniform luminance compared to the external light source used in the traditional nasopharyngoscope (*p* < 0.001). However, we consulted with an ENT practitioner to discuss this disparity, and the luminance of both devices was ruled to be acceptable during practice.

#### 3.2.7. Grayscale Analysis

**Figure 13:**
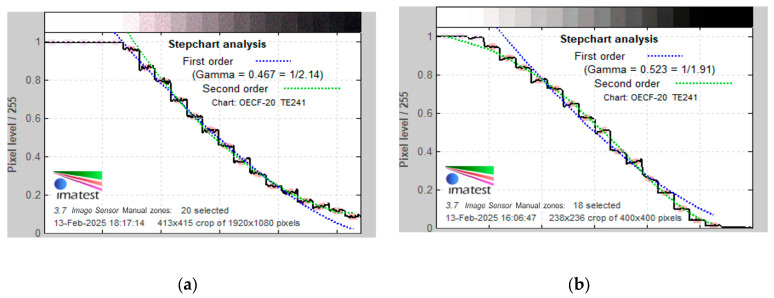
Grayscale stepchart analysis for both the (**a**) traditional nasopharyngoscope and (**b**) NasoLens. The *x*-axis represents the color scale from white to black, while the *y*-axis represents the pixel level. The black line depicts the average normalized pixel level displayed at each patch, while the dashed blue and green curves display the first and second order density fits, respectively [26]. The traditional nasopharyngoscope struggles to discern grayscale in whiter areas, as the pixel levels are the same for the first five scales.

Grayscale stepcharts captured by both endoscopes were analyzed. The results revealed that the traditional nasopharyngoscope performed better in dark areas but struggled in white areas, as it could not distinguish between the first five scales. In contrast, NasoLens displayed a more balanced performance, struggling with only the final two scales of the dark area. The traditional nasopharyngoscope distinguished 16 of the 20 different grayscales, whereas NasoLens was able to identify 18 scales. The results showed that NasoLens demonstrated superior discernment of grayscale, as illustrated in Figure 13.

### 3.3. Result of IRB Testing of NasoLens’ Ability to Allow Sleep

Of the 11 participants, 9 (81.8%) were able to naturally fall asleep while wearing NasoLens, as shown in Table 2. These participants demonstrated stable sleep onset and maintenance throughout the procedure, with consistent readings from the sleep sensors indicating normal sleep cycles. The remaining two participants reported difficulty falling asleep, but this was primarily attributed to discomfort from the home PSG equipment rather than NasoLens itself. The NasoLens device, which was well-tolerated by the majority of participants, did not inhibit the ability to fall asleep. No adverse events were recorded during the trial and participants generally reported the device as comfortable to wear.

## 4. Discussion

### 4.1. Discussion of NasoLens Safety and Effectiveness Simulation

The results indicate that the NasoLens device, with its smaller dimensions and flexible design, can effectively navigate the nasal passage to reach key anatomical sites necessary for sleep endoscopy while minimizing discomfort. By reducing contact with sensitive structures, such as the nasal mucosa and posterior pharyngeal wall, NasoLens allows for a more comfortable procedure compared to larger endoscopes. This feature is particularly beneficial in non-anesthetized procedures, where patient comfort is critical. The device’s ability to capture clear and accurate images of critical areas like the soft palate and epiglottis suggests that it can provide high-quality diagnostic views necessary for evaluating upper airway obstruction in sleep-disordered breathing. These findings support the potential use of NasoLens as a viable alternative for non-anesthetized sleep endoscopy, offering a safer and more comfortable experience for patients.

### 4.2. Discussion of Comparison of NasoLens to Traditional Nasopharyngoscope

The study demonstrates that NasoLens has the potential to allow natural sleep during a sleep endoscopy procedure, providing a minimally invasive option for non-anesthetized monitoring of sleep patterns and airway function. NasoLens’ compact design and simple navigation played a significant role in minimizing discomfort and allowing participants to sleep without significant disruption.

Its enhanced image processing abilities, on top of its integrated features, such as the built-in LED light source and microphone, also allow for improved diagnostic effectiveness. These findings suggest that NasoLens’ design offers an effective alternative to traditional methods that often require anesthesia, which could improve patient outcomes. The ability of NasoLens to facilitate natural sleep also offers the potential for more realistic assessments of sleep disorders, providing a more accurate reflection of patients’ sleep behavior in their natural state. Future studies with a larger sample size and further refinement of device comfort and function are needed to fully evaluate NasoLens’ efficacy and feasibility in broader clinical applications.

### 4.3. Discussion of IRB Testing of NasoLens’ Ability to Allow Sleep

The results of this IRB-approved study suggest that NasoLens is capable of facilitating natural sleep in a significant majority of participants without the need for sedatives or anesthesia. The device’s compact size, flexible design, and integrated features, including simple navigation and an LED light source, allowed most participants to fall asleep naturally and maintain sleep throughout the procedure. The 81.8% success rate indicates that NasoLens has the potential to offer a more comfortable, non-invasive alternative to traditional sleep endoscopy methods, which typically require sedation. While a small portion of participants experienced difficulty falling asleep, this was primarily due to the discomfort associated with the home PSG equipment, not the NasoLens device itself. These findings highlight the feasibility of NasoLens for non-anesthetized sleep assessments and suggest that further refinement of the device could improve patient comfort and sleep initiation in a higher percentage of participants.

### 4.4. Implications of Design Features

NasoLens’ compact and ergonomic design was found to be a key factor in its ability to eliminate the need for anesthesia in sleep endoscopy. Its smaller size and teardrop-shaped head allowed for easier navigation through the nasal passages, minimizing discomfort during insertion. Its flexible wire also removes the need for manual navigation, allowing for smoother insertion and straightforward navigation maneuver. The specialized camera angle provided clearer and more accurate visualization, reducing the need for repeated adjustments during the procedure. Additionally, the integrated microphone offered valuable auditory feedback, which could be used to monitor airway sounds and aid in diagnosis.

### 4.5. Value of Avoiding Anesthesia

Anesthesia during sleep endoscopy presents several risks that can complicate both the safety and accuracy of the procedure. One of the main concerns caused by the use of sedatives is excessive respiratory depression, increasing the risk of airway collapse, which may lead to disastrous consequences including hypoxemia, bradycardia, or cardiac arrest when overlooked [27,28,29]. It should be emphasized that OSA patients, who often undergo sleep endoscopy, present an even higher risk of perioperative complications [30]. This may distort the natural airway dynamics and mask the true pathology of obstruction, and can result in an inaccurate assessment of conditions such as obstructive sleep apnea. Another concern is the unclarified extent of agreement between DISE and NSE, on top of the lack of standardization of protocol and classification [4,31,32]. After the procedure, there is also the potential for residual sedative effects such as hypotension or lingering airway obstruction, which may necessitate prolonged observation and monitoring during recovery [32,33]. Anesthesia also increases procedural costs, causing the process to be less accessible and more resource-intensive. Avoiding anesthesia during sleep endoscopy offers significant clinical and patient-centered advantages by addressing these problems. These benefits underscore the transformative potential of NasoLens in enabling safer and more efficient sleep endoscopy, improving diagnostic accuracy and overall patient outcomes.

### 4.6. Potential for Clinical Use

The results of the experiments suggest that NasoLens has the potential to significantly improve the practice of sleep endoscopy by reducing the need for anesthesia. The ability of NasoLens to allow natural sleep in patients without the use of sedatives is a particularly promising feature, as it enhances patient safety, reduces procedural costs, and increases accessibility to the procedure. These findings point to the possibility of NasoLens being used as a standard tool in sleep endoscopy, particularly for patients who are at higher risk for anesthesia-related complications.

### 4.7. Limitations and Future Research

A key limitation of this study is that NasoLens is still a prototype, and while preliminary results from the experiments are promising, it is crucial that a comprehensive evaluation regarding its safety, effectiveness, and regulatory compliance be conducted before it can be used in clinical practice. As a prototype, NasoLens has gone through 10993 biocompatibility testing, including cytotoxicity, sensitization, and irritation (Table 3), but has not yet undergone the full spectrum of testing required for medical devices, and it is essential that it undergoes a series of thorough assessments to ensure it meets all necessary safety standards. These evaluations must include rigorous sterilization, durability, and electrical safety tests to ensure the device is safe for use in clinical settings and does not pose any risks to patients.

Although an IRB has been completed, the small sample size may not fully represent the diverse patient population that would undergo sleep endoscopy. Additionally, the IRB study only considered short-term outcomes, and further research is necessary to assess long-term safety and efficacy. Future studies should focus on larger cohorts, multi-center trials, and real-world clinical settings to validate the findings and establish the broader applicability of NasoLens.

To meet the necessary regulatory requirements, NasoLens must comply with various ISO and IEC standards. Specifically, ISO 10993 is required for biocompatibility testing to assess any potential adverse reactions to the materials used in the device when in contact with human tissues [34]. ISO 11135 will be necessary to validate the sterilization methods for the device, ensuring that it can be properly sterilized without compromising its function or safety [35]. Additionally, ISO 11737 will provide guidelines for microbial testing, confirming that NasoLens can be safely used without posing a risk of infection [36]. Durability and performance evaluations will need to adhere to ISO 13485 and IEC 60601, the latter of which provides requirements for the safety and performance of medical electrical equipment [37,38]. Furthermore, IEC 62304 will be essential for software lifecycle management if NasoLens includes any software components, ensuring the software is safe and functional throughout its usage [39].

In addition to these standards, NasoLens must comply with regulatory requirements for clinical trials, including ethical approvals and adherence to Good Clinical Practice (GCP). Institutional Animal Care and Use Committee (IACUC) approval will be necessary if any animal testing is involved in evaluating the device’s performance or safety. Furthermore, as NasoLens moves closer to clinical application, consideration must be given to its environmental sustainability. This includes evaluating the environmental impact of manufacturing processes, material sourcing, and end-of-life disposal, ensuring that the device is not only safe and effective but also environmentally responsible. Until these comprehensive safety, regulatory, and sustainability evaluations are completed, the potential for NasoLens to be safely and effectively used in clinical practice remains unconfirmed, making further testing and compliance with relevant standards essential for its development.

## 5. Conclusions

NasoLens has shown great potential as a tool to eliminate the need for anesthesia in sleep endoscopy. Its innovative design features, including a reduced size, ergonomic shape, and integrated microphone, contribute to its effectiveness in providing a more comfortable and safe procedure for patients. The ability of NasoLens to allow sleep without anesthesia represents a significant advancement in the field, with the potential to improve patient outcomes, reduce procedural risks, and make sleep endoscopy more accessible. Further studies are needed to confirm NasoLens’ safety and efficacy across a broader patient population and establish its role in clinical practice.

## Figures and Tables

**Table 1:** Specifications of two nasopharyngoscopes. The table compares specifications of NasoLens to the Karl Storz HD Video Rhino-Laryngoscope (KARL STORZ Endoskope^®^, Tuttlingen, Germany).

Feature	Novel Mini-Nasopharyngoscope	Karl Storz HD Video Rhino-Laryngoscope
Endoscope Head Diameter	ø 2.5 mm × 6.4 mm	ø 3.7 mm × 30 mm
Shape of Endoscope Head	Teardrop-shaped for smoother passage and reduced trauma	Flat cylindrical shape
Endoscope Wire Diameter	1.1 mm	3.7 mm
Insertion	Thin flexible insertion wire and ergonomic tip design. Does not require lubricant or anesthesia	Thick insertion section with stiffer insertion material. Requires lubricant and/or anesthesia.
Navigation	Lower learning curve: Simple navigation to target location with free wire manipulation	Higher learning curve: Manual navigation via a guidance wire and control lever: 140° up/down angulation range
Field of View	Larger (120°)	Smaller (100°)
Distortion	Smaller (−11.3%)	Larger (−13.8%)
Resolution	Lower, due to minimized dimensions	Higher
Color Difference	Equal (ΔC: 15.2, ΔE: 21.0)	Equal (ΔC: 16.6, ΔE: 20.1)
Grey Scale	18 Scales	16 Scales
Luminance	Lower (75.6%) using mini-LED	Higher (82.3%) using external light source
Light Source	Integrated LED light to avoid excessive heat and bulky hardware	External fiber optic light source
Microphone	Implemented to record auditory signals correlated to obstruction.	None

**Table 2:** IRB experiment: ability to fall asleep under natural conditions.

Subject	Able to Fall Asleep	Sex
1	Y	M
2	Y	M
3	Y	M
4	N	F
5	N	M
6	Y	F
7	Y	M
8	Y	M
9	Y	F
10	Y	M
11	Y	M

**Table 3:** ISO 10993 evaluation results of endoscope.

Evaluation	Result	Remark
Cytotoxicity	Pass	Cytotoxicity test results showed “Zero” reactivity. Non in vitro cytotoxicity could be considered in the extract solution.
Skin Irritation	Pass	Test article extracts induced neither observable clinical signs nor dermal gross changes on New Zealand White Rabbits.
Skin Sensitization	Pass	Twenty-four and forty-eight hours after the challenge phase, neither the control nor the treatment group showed visible changes in skin response on the treated areas.

## Data Availability

The data used to support the findings of this study are available from the corresponding author upon request. The data are not publicly available due to confidentiality agreements.
